# Does Mental Influence in the Mother Produce Malformation in the Fœtus

**Published:** 1870-08

**Authors:** 


Does Mental Influence in the Mother Produce Malformation in the Fœtus.—The *American Journal of Insanity* contains an article on this subject, by Dr. G. J. Fisher, of which the following is a brief summary:

It is a traditional superstition, for which the medical profession is largely responsible, that malformations are the result of maternal mutual emotions. Various and intense emotions are common in gestating women, as are apprehensious of malformation of their offspring, yet abnormal births are exceedingly rare.

There is nothing like law on the alleged results of maternal mental influences in the production of malformations, and the occasional apparent relation of cause and effect is due, usually, to accidental coincidences, which would be far less frequent if the facts could be ascertained *before* instead of *after* the birth of the child. The coincidences are not sufficiently numerous and authentic to warrant the belief that they are anything else. Like cause produce like results; whereas widely dissimilar emotions are assigned for causes of the same deformity. Some of the assumed causes are alleged to have operated on the fœtus *subsequent* to the time for the evolution of the part deformed. In a large proportion of the malformations no mental or physical explanation is offered by the parents.

The alleged resemblance of maculæ to contain fruits and anim[illegible] is purely accidental or is the result of imagination

Malformations identicle in kind and degree recur often on the human subject, and admit of classification. They all have their exact morphological counterpart in the lower animals, as well as some that are analogous in the vegetable kingdom.

Monstrosities are not the result of *violation* of embryological or physiological laws; they are the product of *embarrassment*, to normal development, either retarded, arrested, or excessive development.

In plural conception it is absurd to suppose mental emotion can be conveyed to one fœtus in the womb and not to the other, whether it be transmitted through the blood, by undiscovered nerves in the *funis*, or by some animal magnetism directly across tissues.

The development of united-embryos is now known to follow definite laws.

Monstrosities may arise from either abnormalities of the generative matter of one or both parents, or of the material organism, or from abnormal states of the membranes of the ovum and umbilical cord.

The time has fully come to repel the popular error that anomalies of organization are due to mental emotions; and it is the duty of the medical profession to aid, as they carry on this consummation.

